# The *PIKE* Homolog *Centaurin gamma* Regulates Developmental Timing in *Drosophila*

**DOI:** 10.1371/journal.pone.0097332

**Published:** 2014-05-20

**Authors:** Anna Lisa Gündner, Ines Hahn, Oliver Sendscheid, Hermann Aberle, Michael Hoch

**Affiliations:** 1 University of Bonn, Life & Medical Sciences Institute (LIMES), Molecular Developmental Biology, Bonn, Germany; 2 Heinrich-Heine-Universität Düsseldorf, Funktionelle Zellmorphologie, Düsseldorf, Germany; CINVESTAV-IPN, Mexico

## Abstract

Phosphoinositide-3-kinase enhancer (PIKE) proteins encoded by the *PIKE/CENTG1* gene are members of the gamma subgroup of the Centaurin superfamily of small GTPases. They are characterized by their chimeric protein domain architecture consisting of a pleckstrin homology (PH) domain, a GTPase-activating (GAP) domain, Ankyrin repeats as well as an intrinsic GTPase domain. In mammals, three PIKE isoforms with variations in protein structure and subcellular localization are encoded by the *PIKE* locus. *PIKE* inactivation in mice results in a broad range of defects, including neuronal cell death during brain development and misregulation of mammary gland development. *PIKE -/-* mutant mice are smaller, contain less white adipose tissue, and show insulin resistance due to misregulation of AMP-activated protein kinase (AMPK) and insulin receptor/Akt signaling. here, we have studied the role of PIKE proteins in metabolic regulation in the fly. We show that the *Drosophila PIKE* homolog, *ceng1A*, encodes functional GTPases whose internal GAP domains catalyze their GTPase activity. To elucidate the biological function of *ceng1A* in flies, we introduced a deletion in the *ceng1A* gene by homologous recombination that removes all predicted functional PIKE domains. We found that homozygous *ceng1A* mutant animals survive to adulthood. In contrast to *PIKE -/-* mouse mutants, genetic ablation of *Drosophila ceng1A* does not result in growth defects or weight reduction. Although metabolic pathways such as insulin signaling, sensitivity towards starvation and mobilization of lipids under high fed conditions are not perturbed in *ceng1A* mutants, homozygous *ceng1A* mutants show a prolonged development in second instar larval stage, leading to a late onset of pupariation. In line with these results we found that expression of ecdysone inducible genes is reduced in *ceng1A* mutants. Together, we propose a novel role for *Drosophila* Ceng1A in regulating ecdysone signaling-dependent second to third instar larval transition.

## Introduction

Centaurins comprise a family of multidomain proteins that regulate a variety of cellular processes including cell survival, cell cycle progression, cell migration, receptor and endosome trafficking, gene transcription as well as development of dendrites and synapse conductivity [1]–[4]. Misregulation of their expression and defects in function have been associated with Alzheimer's disease [5] and a number of cancers, such as glioblastomas, sarcomas or neuroblastomas (reviewed in [6]–[9].

Centaurins were named after the centaurs of Greek mythology due to their chimeric protein domain architecture. They contain a pleckstrin homology (PH) domain mediating membrane recruitment and a GTPase-activating (GAP) domain catalyzing hydrolysis of GTP on ADP-ribosylation factor (Arf) proteins. Ankyrin repeats at the C-terminus potentially mediate protein-protein interactions. In addition, members of the Centaurin gamma subfamily harbor an intrinsic GTPase domain whose activity can be modulated by the GAP domain, enabling them to act as molecular switches [7].

In mammals, the Centaurin gamma homolog is encoded by the *PIKE*/*CENTG1* gene from which three protein isoforms, termed PIKE (Phosphoinosite 3-kinase enhancer)-A, L, and -S are produced through alternative splicing from a single gene locus [7], [8]. PIKE-L is the longest isoform and contains a proline-rich domain at its N-terminus [10]. PIKE-S lacks both the ArfGAP domain and the C-terminal ankyrin repeats [11]. PIKE-A resembles PIKE-L apart from the N-terminus: the N-terminal proline-rich domain of PIKE-L is replaced by a short peptide [12].

A PIKE mouse model lacking the conserved domains of all three PIKE isoforms revealed a function of PIKE in metabolic control in peripheral tissues [13]. *PIKE^−/­−^* knockout mice are resistant to diet-induced obesity, show reduced white adipose tissue, a decrease in adipocyte formation, enhanced lipid oxidation and increased insulin sensitivity. In vitro, an interaction between PIKE-A and the insulin receptor negatively regulates the activity of AMP-activated protein kinase (AMPK), the master sensor of energy status suggesting that PIKE-A is implicated in obesity and associated diabetes development by modulating AMPK activity [13].

Apart from the regulation of metabolism in peripheral tissues, PIKE proteins have critical functions in the nervous system, where PIKE-S and –L mainly carry out antiapoptotic and neuroprotective functions by linking various signaling pathways to PI3K/Akt signaling [14]. PIKE-L serves as a link between the type I metabotropic glutamate receptors (mGluRs) and the PI3K-dependent cell survival signal in neurons [15]. Furthermore, PIKE-L is involved in netrin-dependent processes: by binding the netrin receptor UNC5B, PIKE-L activates the neuronal PI3K pathway upon receptor activation, promoting neuronal survival [16]. Upon nerve growth factor (NGF) induction, PIKE-S augments PI3K activation in the nucleus leading to the induction of cyclinD1, thereby translating the NGF-dependent mitogenic signal to the nucleus [17], [11].

In summary, the Centaurin gamma/PIKE subfamily of proteins has diverse functions in different tissues. To gain more insight into this conserved family of proteins, we made use of the model *Drosophila melanogaster*. Here we show that the single homolog of *CENTG1* in *Drosophila*, *centaurin gamma 1A* (*ceng1A*), encodes for a functional GTPase whose GTPase activity is catalyzed by the internal GAP domain. Furthermore, we generated *ceng1A* mutants by removing all conserved domains via homologous ends-out gene targeting. Our analysis revealed that the function of Ceng1A in *Drosophila* does not recapitulate the PIKE dependent regulation of metabolic control in peripheral tissues seen in mouse but affects timing of larval development. Second instar larval stage is prolonged in the mutants coinciding with a reduced growth rate during this stage. Our results suggest that Ceng1A plays a previously not described role in regulating developmental timing independent of nutrient conditions, resulting in reduced ecdysone signaling.

## Materials and Methods

### Fly stocks

Flies were raised on standard fly food at 25 °C if not indicated otherwise. The following fly stocks were used: wild-type (*white^−^*).

To generate a transgenic *UAS-cenG-HA* line, the ORF of *cenG-RA* was cloned into *pUAST* using primers UAS-cen-F1 (GAACACGTCGCTAAGCGAAAG) and UAS-cen-R1 (CGCACTTATTGGCTTCCTCTG). Transgenic flies were generated via transposase-mediated P-element insertion.

### Generation of a *ceng1A* knockout allele

**Figure 1:**
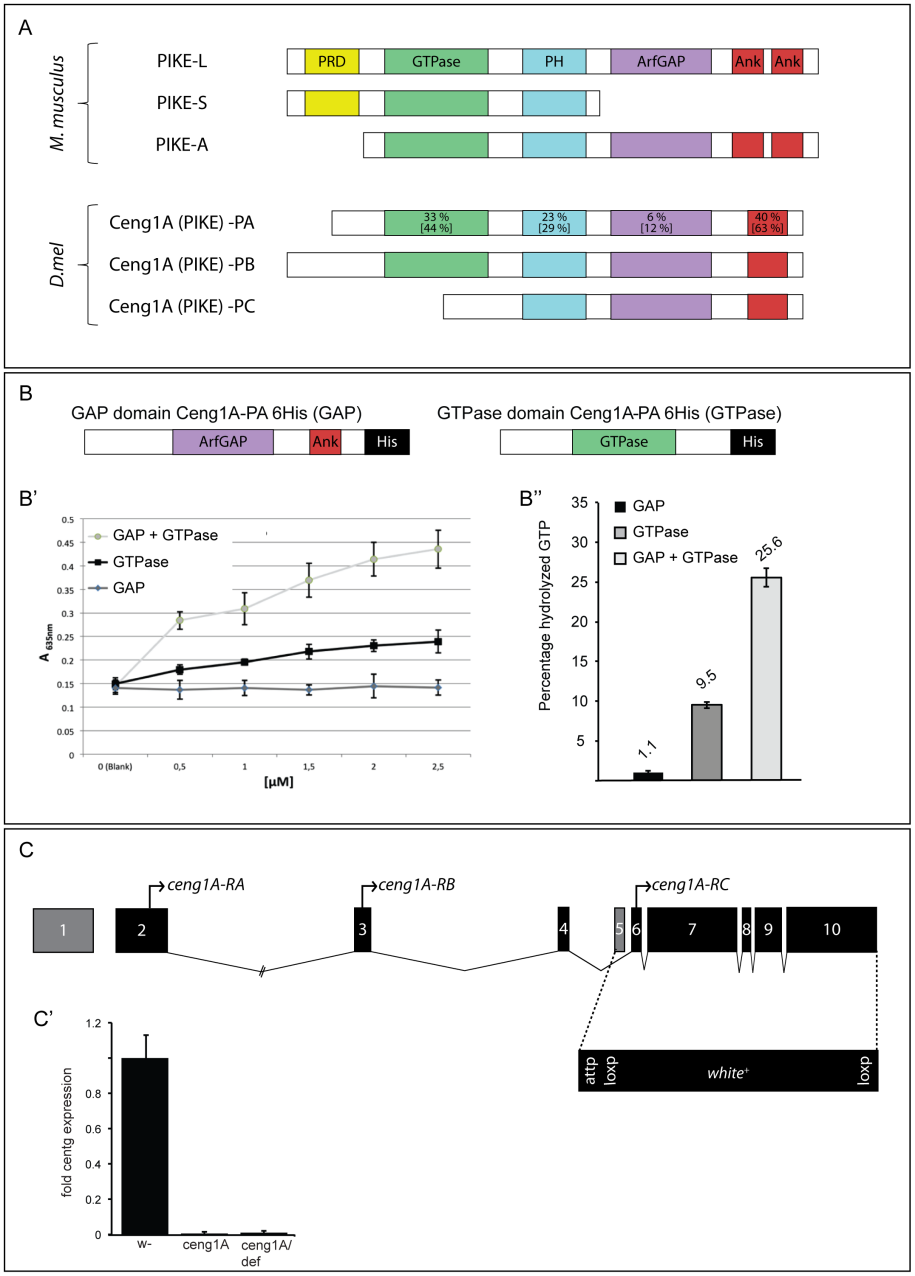
*Drosophila* Ceng1A is a functional GTPase with a catalytic internal GAP domain. (A) Schematic representation of the predicted domain structure of the three murine PIKE isoforms (PIKE-A, -L and –S) and the homologous *Drosophila* Ceng1A isoforms (Ceng1A-PA, -PB and -PC). *ceng1A* codes for a N-terminal GTPase domain, a PH domain, a GAP domain and an ankyrine motif. Predicted domains of the three *Drosophila* Ceng1A proteins with PIKE domains show a high degree of conservation. Numbers indicate percentage of identity [similarity] on the amino acid level. (B) The GTPase and GAP domains of Ceng1A were used for a colorimetric in vitro GTPase assay. Two constructs were cloned and expressed in *E. coli* for the analysis: A 6xHis tagged construct containing the C-terminus of Ceng1A including the GAP domain and a 6xHis tagged construct containing the GTPase domain. (B') The graph depicts absorption at 635 nm versus protein concentration in µM. Addition of the GAP domain increases GTP hydrolysis of the GTPase domain. (B’’) Relative amount of hydrolyzed GTP. The GTPase domain alone shows modest GTPase activity, but activity was increased 1.5-fold by including the GAP domain. (C) Gene locus organization and generation of *ceng1A* knock-out mutants. Exon/intron structure of the *ceng1A* locus is depicted. Start sites of the three transcripts (*ceng1A* -RA, -RB and –RC) are indicated. A loss-of-function mutation for *ceng1A* was generated by ends-out gene targeting [19]. The targeting construct for the homologous recombination was designed to delete exons 5–10, which encode all functional domains. (C') *Real-time* RT-PCR analysis of *ceng1A* expression in the generated *ceng1A* mutants.

To generate a *ceng1A* mutant allele, a gene targeting approach was performed according to the modified ‘Rong and Golic’ protocol [18]. Gene targeting was designed in a way that exons 5 to 10 of the *ceng1A* ORF (harbouring the conserved domains) were deleted. The 5′ homology arm (using primers GAATTCAATGAGCCATAGTTTCCTCCCTT and GCGGCCGCTCCACTTAACAAGTACCGTGCATTA) and 3′ homology arm (using primers GGCGCGCCGTGAGGTACAGTGTCGATGTAAAGC and CCTAGGGAGTCTGCGAGTTGCCTTTTAAG) were cloned into pRK2. Targeting, screening and balancing crosses were performed as described in [19]. Candidates were verified via a PCR test strategy (see Figure 1C). Knockout of *cenG* was tested via *real-time* RT-PCR (Figure 1C).

### Quantitative *real-time* RT-PCR

For RNA isolation, at least ten *Drosophila* third instar larvae or 20 second instar brains were placed in lysis buffer and homogenized using a Precellys tissue homogenizer. Total RNA was isolated using the NucleoSpin RNA II kit (Macherey & Nagel) and RNA concentration was analyzed via a NanoDrop spectrophotometer (Thermo Scientific). For first strand cDNA synthesis, 500 ng of total RNA was transcribed using the QuantiTect Reverse Transcription Kit (Qiagen) including DNaseI treatment following the supplieŕs protocol. *Real-time* PCR was performed with 1 µl cDNA per reaction using the IQ SYBR Green Supermix (Biorad) as detection dye. Experiments were performed with the iQ5 *Real-Time* PCR Detection System from BIO-RAD. cDNA samples were run in triplicates, the average C_T_ was used to analyze the expression levels via the -2^ΔΔCT^ method. Experiments were repeated with independently isolated RNA samples. *Actin 5C* (*Act5C*, *act*) and *Ribosomal protein L32* (*RpL32*, *rp49*) were used as reference genes. Expression Analysis was performed using BIO-RAD iQ5 Optical System software (version 1.1.1442.OCR), following the instruction provided by the supplier and Microsoft Excel. The following oligonucleotides were used for *real time* PCR analysis: see Table 1.

**Table 1:** *real-time* RT-PCR primers.

Gene	Forward primer	Reverse Primer
*rp49*	CTAAGCTGTCGCACAAATG	GTTCGATCCGTAACCGATGT
*act*	GTGCACCGCAAGTGCTTCTAA	TGCTGCACTCCAAACTTCCAC
*cenG1A*	AGGAAGAACGTAACCGGAGCAG	AGGTAGTGCCCAATCTTGACCAG
*lip3*	TGAGTACGGCAGCTACTTCCCT	TCAACTTGCGGACATCGCT
*4EBP*	CATGCAGCAACTGCCAAATC	CCGAGAGAACAAACAAGGTGG
*E74A*	TGTCCGCGTTTCATCAAGT	GTTCATGTCCGGCTTGTTCT
*E75B*	CAACTGCACCACCACTTGAC	GCCTTGCACTCGTTCTTCTC
*PTTH*	GAGCTGCAGAAGGAGTACAG	CGATTCCTTTATTTGTAGGCT

### Weight, size and wing analysis

Weights of adult flies were determined using an analytical balance: ten seven-days old male or female flies were anesthetized and weighed in a 1.5 ml tube. Sizes of adult flies were determined using Olympus SZ12 binocular and ImageJ for quantification. For wing size measurements, flies were anesthetized, and wings dissected and placed on a slide with a drop of water. Wings were imaged with an Olympus SZ12 binocular via SIS Analysis 2.1 software. Relative wing areas were analyzed in ImageJ (National Institute of Health).

### GTPase assay

Two constructs where cloned into pTriEx: a construct harboring the N-terminus of Ceng1A-PA including the GTPase domain (Ceng1A-PA GTPase) as well as construct containing the C-terminus of Ceng1A including the GAP domain. The constructs were expressed in *E. coli* and purified via their His-tags. GTPase activity was analyzed using the GTPase Assay Kit (Innova Bioscience) according to the manusfacturer's instructions. Samples were analyzed in a 96 well plate reader at 590–660 nm wavelength.

### Triacylglyceride assay

Ten male or female flies were homogenized in 250 µl methanol/chloroform mixture using a Precellys tissue homogenizer. Samples were analyzed in triplicates. Five µl of each lysate was applied on a silica gel plate (carrier material: aluminum). A 4∶1 hexane/ethylether mixture was used as mobile phase. After sample runs, the plate was incubated in detection solution (15.6 g copper sulfate pentahydrate, 9.6 ml phosphoric acid ad 100 ml H_2_O) and incubated at 180°C for 10 minutes. Butter was used as TAG standard.

TAG measurements of starved flies were conducted after 0, 20 and 28 hours. Flies on high fat diet were analyzed after 7 days on the respective food conditions (standard or high fat).

### OilredO staining

Larval fat bodies were dissected in PBS and fixed for 1 hour in 4% PFA. Samples were washed twice with H_2_O and afterwards stained for 30 minutes with oilredO (6 ml Oil Red O stock solution (0.06% Oil Red O in isopropanol). Specimens were washed twice with H_2_O, embedded in Fluoromount and immediately imaged.

### Survival analysis

Twenty-four-hour old male or fertilized female flies were placed on standard food in groups of ten. After 10 days on standard food, flies were transferred on normal or starvation conditions (0.8% agarose in PBS). For each experiment, 5×10 flies were analyzed for both conditions. Flies on starvation conditions were checked every four hours. Data were analyzed using Xlstat. Survival curve and average survival rate were determined with the Kaplan-Meier-estimator. Logrank test was used to determine statistical significance.

### Feeding assay

The feeding assay was modified from Xu et al [20]: Early L2 stage control and *ceng1A* mutant larvae were fed with Brilliant Blue FCF colored yeast for 6 hours. After the feeding, weight of larvae was measured. Larvae were homogenized in 200 µl PBS buffer and centrifuged (13000 rpm) for 30 min. The supernatants were transferred to a new tube and centrifuged again at 13000 rpm. Absorbance was measured at 625 nm. The absorbance measured for supernatants from flies fed with normal food was subtracted from the absorbance of the supernatant from blue food-fed flies. Food intake was depicted as absorbance relative to weight.

### Western blots

Early third instar larvae were starved for four hours or kept on yeast agar plates, respectively. Approximately 60 larvae per condition and genotype were pooled and RIPA lysis buffer containing phosphatase inhibitors was added according to weight (2 µl buffer per mg larvae). Larvae were homogenized, cooked at 75°C for 10 min, centrifuged for 20 min and 10 µl per sample was immediately used for the SDS page and subsequent blotting. The following antibodies were used: anti-pAMPK (1∶1000, Cell Signaling #2535), anti-pAkt (1∶2000, Cell Signaling #4060) and anti-actin (1∶1000, abcam # ab1801).

## Results and Discussion

### The conserved *Drosophila* gene *ceng1A* encodes for a functional GTPase with a catalytic internal GAP domain

Similar to murine CENG1/PIKE, it is predicted that the single *Drosophila* CENTG1 homolog *ceng1A* codes for three transcripts (*ceng1A-RA*, *RB* and *–RC*). We validated *ceng1A-RA* and –*RB* expression in third instar larvae via transcript specific RT-PCR and subsequent sequencing. Both Ceng1A-PA and -PB code for a GTPase domain but all three variants contain the PH domain, the ankyrin repeats and the ArfGAP domain (Figure 1A).

All three mammalian CENTG1 proteins (PIKE-A, -L and –S) have been shown to bind GTP and exhibit GTPase activity [21], [22]. In addition, for CENTG1 and CENTG2 it has been demonstrated that the C-terminal GAP domain can stimulate the internal GTPase activity [21]. To test whether Ceng1A acts as a functional GTPase and whether its GAP domain can catalyze GTPase-dependent GTP hydrolysis, we performed a colorimetric GTPase assay. The assay is based on a photometrically detectable complex of free inorganic phosphate and a dye (P_i_ ColorLock Gold reagent, Innova Bioscience). We used the following constructs: Centg1A-PA GTPase (GTPase), harboring the N-terminal GTPase domain as well as Centg1A-PA GAP (GAP) containing the C-terminal GAP domain (Figure 1B).

In our assay we observed a Ceng1A-PA GTPase concentration-dependent increase in GTP hydrolysis, which could not be seen in an approach just using Ceng1A-PA GAP (Figure 1B',B’’). Adding the GAP domain (Ceng1A-PA GAP) to Ceng1A-PA GTPase increased GTP hydrolysis (Figure 1B'): whereas the GTPase domain alone shows only a low ability to hydrolyze GTP (9.6% of the total GTP were hydrolyzed), addition of the GAP domain increased hydrolysis by factor of 1.5 (25.6% hydrolyzed GTP; Figure 1B’’). This indicated that the GTPase domain of Ceng1A is able to hydrolyze GTP in vitro. Furthermore, increased hydrolysis in the presence of the GAP domain shows that Ceng1A is a GTPase, which is catalyzed by its intrinsic GAP domain.

### Ceng1A is predominantly expressed in the CNS

In mammals, the three PIKE isoforms show distinct expression patterns. Whereas PIKE-A is expressed ubiquitously, PIKE-S and –L isoforms are both specifically expressed in the nervous system [4].

*ceng1A* is expressed at moderate levels throughout development in a rather ubiquitous pattern [23]. We analyzed the *ceng1A* expression pattern via in-situ hybridization in embryonic and larval stages. The probe detecting exons 5 to 10 of the *ceng1A* ORF recognized overexpressed *ceng1A* (Figure S1I) and did not show a signal in the mutants (Figure S1J), allowing the conclusion that the synthesized probes recognize *ceng1A* specifically.

The earliest stage where *ceng1A* expression could be detected was in the cellular blastoderm: Here, *ceng1A* is weakly expressed in a ubiquitous fashion with a strong enrichment in the anterior ectoderm (Figure S1A). From stage nine onwards, *ceng1A* is expressed in the developing nervous system: neuroblasts in the head region as well as in the ventral nerve cord show a signal (Figure S1B). Furthermore, parts of the midgut are stained. In later stages *ceng1A* can be detected in the areas of the central (the brain and the ventral nerve cord) as well as in the peripheral nervous system (sensoric head complex, peripheral sensory clusters; Figure S1C-E).

In larval stages, the only tissue where we could detect *ceng1A* expression was the brain (Figure S1G). In contrast to embryonic stages, there was no signal detectable in the gut (Figure S1H). The highest levels of *ceng1A* expression were in the optic lobes of the CNS, with enrichment in two lateral stripes corresponding to the outer proliferative center (OPC) the precursors of the optic lobes. Overall, *ceng1A* expression can mostly be found in tissues of the nervous system.

### Generation of a *ceng1A* mutant

In order to analyze the *in vivo* function of the *ceng1A* gene products we generated loss-of-function mutants using ends-out gene targeting as described by [18] and [19]. The *ceng1A* gene locus consists of ten exons which are spread across 60 kb of chromosome 2L. Three transcript variants are predicted, which differ in the transcriptional start sites (Figure 1C). All transcripts share the last six exons (exons 5 to 10).

We targeted the *ceng1A* locus in a similar fashion as was described for the *PIKE* locus [13] resulting in the removal of all crucial domains: The donor construct for homologous recombination was designed to delete exons 5 to 10. We were not able to target the entire open reading frame of *ceng1A*, which would have generated a null allele for all transcripts, since six independent genes are located in between exon one and two of *ceng1A*. By targeting exons 5 to 10, we deleted most of the GTPase (63%) plus all the following conserved domains (Figure 1C). The genomic sequence was replaced by a targeting construct carrying a *white^+^* marker gene and a Φ31 integration site (attp site) [19]. *White^+^* positive candidates were recovered from the screening cross and tested by genomic PCR analysis (Figure S2A'). We identified two independent lines with precise insertion of the construct. *ceng1A* mutants lack exons 5–10 and loss of *ceng1A* expression was further confirmed by *real-time* RT-PCR analysis: no expression of *ceng1A* could be detected in either homozygous mutants or in animals carrying the mutation over a deficiency allele (Figure 1C'). Furthermore, we found the expression of neighboring genes not to be affected in the mutants (data not shown). Thus, generation of a *ceng1A* null mutant was successful.

### Ceng1A does not have a major impact on metabolic control

**Figure 2:**
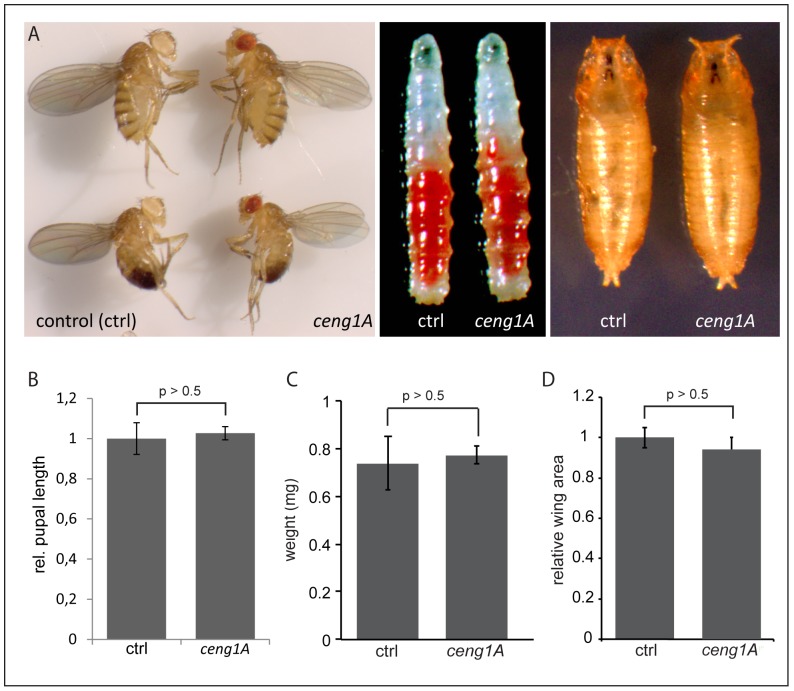
*ceng1A* mutants do not differ from control animals in size and weight in larval, pupal or adult stages. For all experiments zygotic and maternal *ceng1A* mutant animals were used. (A – A’’) Comparison of *ceng1A* mutant adults, larvae and pupa with wildtypic counterparts does not reveal any morphological defects. (B) Length of third instar *ceng1A* mutant pupae is not altered relative to controls. (C) Total weight (in mg) as well as area of the wings (D) does not differ in adult *ceng1A* mutants compared to controls. n = 50; error bars indicate SEM; p>0,5 (t-test).

Similar to the *PIKE* -/- mutants, *ceng1A* mutants did not reveal any obvious morphological defects [13]: homozygous *ceng1A* as well as *ceng1A*/def mutants (data not shown) are viable and fertile (Figure 2A). *PIKE* knockout mice are lighter and smaller compared to their wildtypic counterparts. We analyzed size and weight of wildtypic and *ceng1A* mutant animals. Adult, larval as well as pupal *ceng1A* mutants did not show any differences in size compared to wildtypic controls (Figure 2A, B). In addition, *ceng1A* mutants did not differ in weight (Figure 2C). To assess whether organ size might be affected in *ceng1A* mutants we measured the wing area of *ceng1A* mutants in comparison to *w^−^* flies. Here, the relative *ceng1A* wing area does not differ from control flies (Figure 2D) indicating that weight and size of *ceng1A* mutants is not affected under normal feeding conditions, in contrast to the *PIKE* -/- mice.

**Figure 3:**
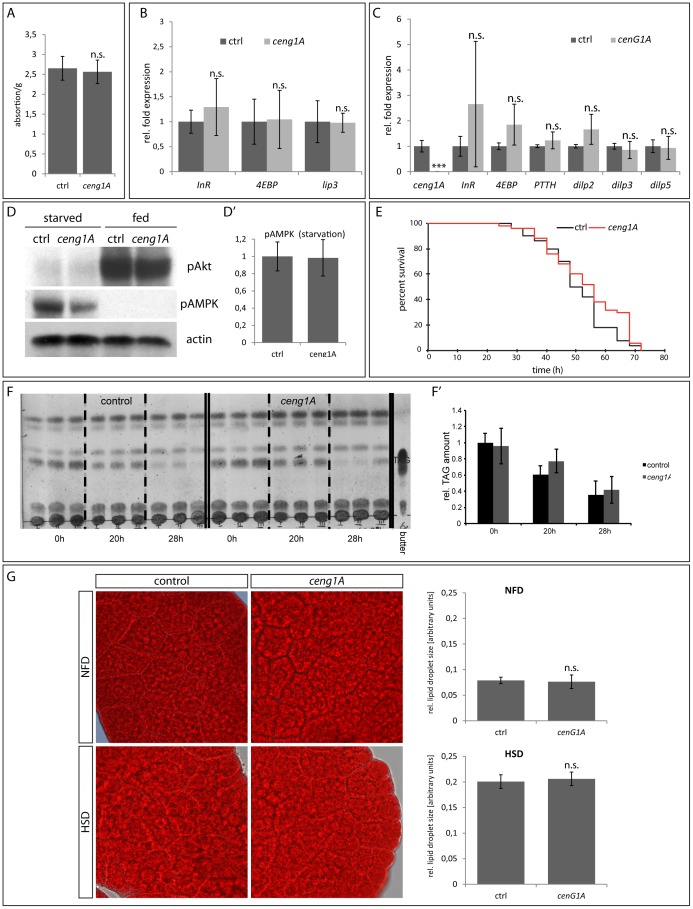
. **Ceng1A is not involved in metabolic control.** (A) *ceng1A* mutant larvae feed normally, since quantitative analysis of Brilliant Blue FCF-colored yeast uptake shows no difference between control and *ceng1A* mutants. n = 10, each n equals 10 larvae; error bars indicate SEM. *Real-time* RT-PCR analysis of early third instar larvae (B) and dissected CNS (C) shows that transcription of *InR*, *4EBP*, *lip3, dilp2, 3* and *5* as well as *PTTH* is not changed in *ceng1A* mutants. n = 10; error bars indicate SEM; n.s.  = p>0,5 (t-test). (D) Immunoblots using anti-pAMPK and anti-Akt antibodies indicate that there is no difference in AMPK and Akt phosphorylation between control and *ceng1A* mutant larvae in starvation and fed conditions. Anti-actin served as a loading control. Quantification (D') of western blots of control and *ceng1A* mutant larvae stained for pAMPK relative to loading control. n = 3; error bars indicate SEM. (E) Survival curve of *ceng1A* mutant and *w-* control flies under starvation conditions. The mean survival time of *ceng1A* mutants (53,6 h) is not significantly changed compared to *w-* control flies (50,5h). p>0.5 (log rank test) (F) Thin layer chromatography to determine triacylglyceride (TAG) levels of control and *ceng1A* mutant flies upon starvation. Height of TAGs is shown in the right lane. Samples were taken at the start of the experiment (0h) and after 20 and 28 hours of starvation. (F') Body TAG levels are not changed in *ceng1A* mutants. Graph represents the relative absorbance of TAG bands quantified by photo densitometry. n = 3; error bars indicate SEM (G) Oil Red O staining of third instar fat bodies. Larvae were grown on either standard food (NFD) or high-sugar food (HSD). Fat body storage lipid load of *ceng1A* mutants does not differ from control larvae. (G') Analysis of relative lipid droplet size reveals no differences between control and *ceng1A* mutant larvae under both conditions. n = 50; error bars indicate SEM; p>0,5 (t-test).

To verify that potential phenotypes of *ceng1A* mutants were not due to feeding defects, we quantified the amount of food intake using a modified assay previously described by Xu et al ([20]; see Material and Methods): Food intake of *ceng1A* mutants is not altered compared to control animals (Figure 3A).

*PIKE* -/- mice showed increased insulin sensitivity [13]. To assess the activity of insulin-like signaling (IlS) in *ceng1A* mutants we analyzed expression of the major downstream targets of the insulin/TOR signaling network, the *eIF4E binding protein* (*4EBP*) as well as the *insulin receptor* (*InR*). *4EBP* and *InR* expression is upregulated under unfavorable conditions by the transcription factor FoxO. When insulin signaling transmission is blocked, like in *steppke* mutants, *4EBP* and *InR* levels are elevated independent of the feeding status [24]. Therefore we would expect that the FoxO targets *4EBP* and *InR* are downregulated if insulin sensitivity is increased. We analyzed FoxO target gene expression in third instar *ceng1A* mutant larvae and compared it to *w^−^* control larvae: Under standard feeding conditions, expression of *4EBP* and *InR* was similar in both genotypes (Figure 3B). Since *ceng1A* is primarily expressed in the nervous system, we also checked whether IlS target genes are affected in those tissues. To that end we dissected early third instar larval brains and checked expression of *4EBP* and *InR* as well as the *Drosophila* insulin-like peptides *dilp2*, *3* and *5* (that are produced in special neurons in the CNS). For none of the genes we tested, expression was significantly altered in *ceng1A* mutants compared to wildtypic animals (Figure 3C).

In a next step we checked if Ceng1A affects metabolic control on protein levels: Two of the major sensors for the nutritional status are AMPK and Akt: We found that AMPK phosphorylation under starvation conditions is still possible in *ceng1A* mutants (Figure 3D, for quantification see Figure 3D', S3). Furthermore, Akt phosphorylation is equally increased in *ceng1A* mutant and control animals under normal feeding conditions (Figure 3D).

IlS and AMPK exert an important function under unfavorable conditions. Increased sensitivity towards IlS leads to a limited capability to cope with nutrient restricted conditions [25]. Mutants with increased sensitivity towards IlS or AMPK mutants are not able to adapt to nutrition-limited conditions and therefore do not reduce their metabolic rate, leading to a rapid exhaustion of fat stores. This results in decreased survival of those mutants compared to their wildtype counterparts raised under the same conditions. To test if *ceng1A* mutants are sensitive to such stress conditions, we maintained five-day old adult flies on nutrient depleted media. The survival time of *ceng1A* mutants, however, was not changed significantly (Figure 3E).

In summary, our results indicate that Ceng1A does not have a major impact on IlS or AMPK signaling.

Since growth, survival or IlS-dependent target gene expression were not affected in *ceng1A* mutants, we tested whether Ceng1A is required for metabolic control, in a manner similar to PIKE-A, which involves regulation of fat storage and mobilization: *PIKE* whole body knockout mice are leaner and display a significant reduction in white adipose tissue and an increase in β-oxidation [13]. We investigated body fat content in *ceng1A* mutant flies using thin layer chromatography. We measured whole body triacylglyceride levels at three different time points during the starvation experiment. Neither at the beginning (0h) nor during the starvation period (20h and 28h) *ceng1A* mutants showed obvious body fat mass differences compared to control flies (Figure 3F, F'). Consequently, we did not observe an induction of *lipase3* expression, another starvation marker, under normal feeding conditions (Figure 3B), indicating that lipid mobilization is not altered in the mutants.

*PIKE ^−^* ^/*−*^ mice are resistant to high-fat diet-induced obesity due to inhibited adipocyte differentiation [13]. We analyzed fat tissue morphology of *ceng1A* and *w^−^* larvae under normal and high-fat conditions. To this end, larvae were grown on standard or high-fat diet and third instar fat bodies were isolated and stained with Oil Red O. Under both conditions no difference in fat body morphology or lipid droplet storage could be observed in *ceng1A* mutants compared to *w-* larvae. Furthermore, lipid droplet size is not altered in *ceng1A* mutant fat bodies compared to controls under both feeding conditions (Figure 3G) indicating lipid storage is not affected. In summary, loss of *ceng1A* does not seem to have an impact on body fat mass or on resistance to high fat diet-induced obesity in flies.

We conclude from these experiments that Ceng1A does seem to play a major role in metabolic regulation in peripheral tissues, in contrast to what was described for its murine homologue PIKE-A.

### Ceng1A regulates developmental timing

**Figure 4:**
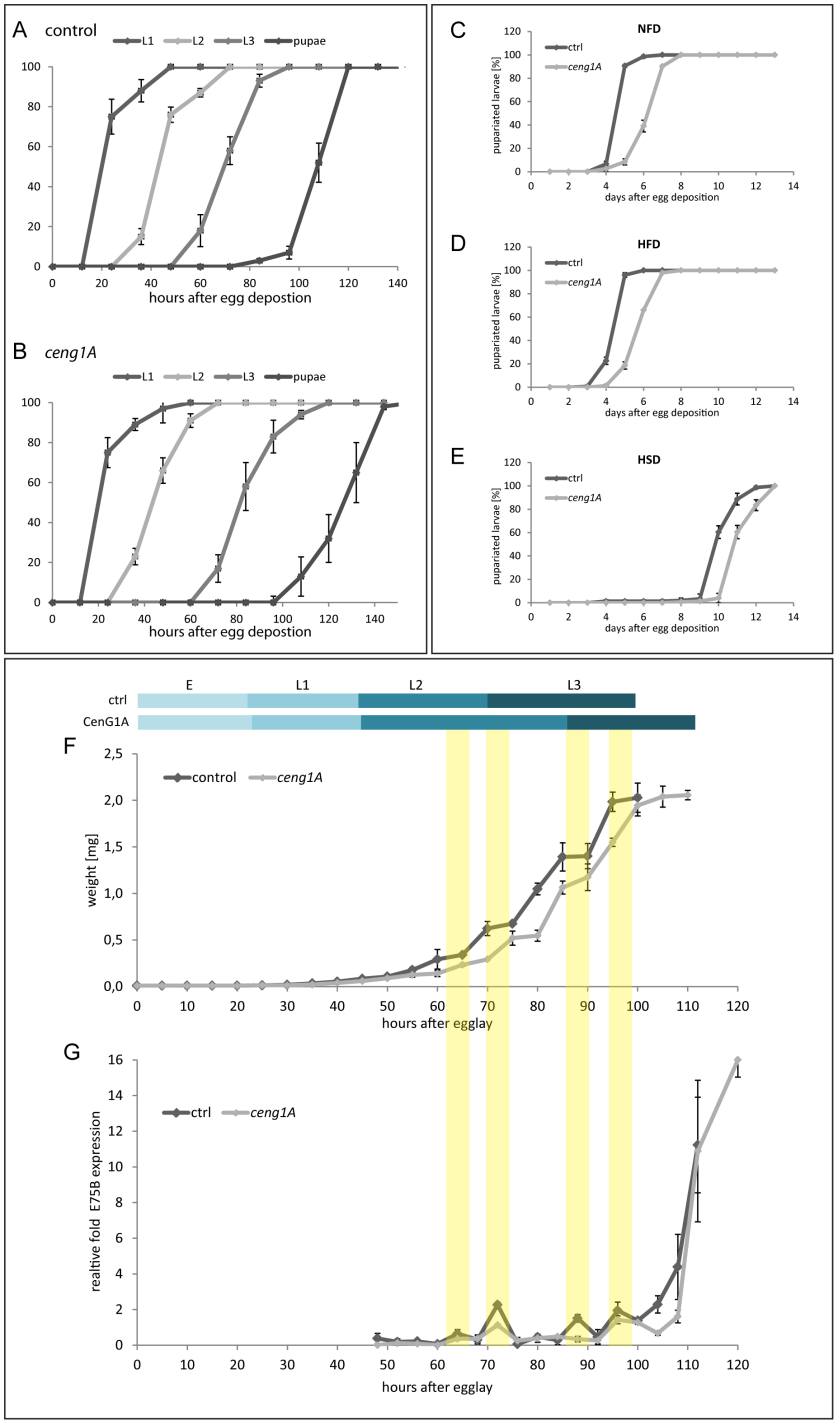
*ceng1A* mutants show delayed second instar larval stage and reduced ecdysone signaling. Relative amount of control (A) and *ceng1A* mutants (B) in L1, L2, L3 and pupal stage was determined from egg lay (0) to 140 hours after egg deposition. n = 3; each n corresponds to 50 larvae; error bars indicate SEM. *ceng1A* mutants are delayed in second instar larval stage compared to controls. (C – E) Onset of pupariation *ceng1A* mutant or wildtypic larvae on standard food (C, NFD), high fat diet (D, HFD) and high sugar diet (E, HSD) was analyzed. Delay in development of *ceng1A* mutant larvae is nutrition independent. n = 3; each n equals 50 larvae; error bars indicate SEM (F) Scheme of average stage length in control and *ceng1A* mutant larvae (derived from A and B). Growth rate was assessed as an increase of weight over time from egg deposition to pupariation. The main delay of growth is between 45 and 80 hours after egg lay (corresponding to second instar larval stage). Afterwards, growth rate of *ceng1A* mutants increases in parallel to the control growth rate, only with the in L2 stage accumulated time delay. (G) From 48 hours after egg deposition to pupariation, expression of the ecdysone target genes *E75B* was analyzed via *real-time* RT-PCR. Peaks of *E75B* expression coincide in control and *ceng1A* mutant animals. However, *E75B* induction levels are reduced in *ceng1A* mutants. Transparent yellow bars correlate peaks of *E75B* expression with the growth rate at that time point. n = 5 for all experiments; error bars indicate SEM.

During our detailed phenotypical analysis of *ceng1A* mutants we noticed a delay in development: Whereas timing of embryonic and first instar development is mostly unaffected in *ceng1A* mutant animals, the second instar larval stage is prolonged leading to a delayed onset of pupariation (Figure 4A, B). The duration of the L3 stage seems not to be affected (Figure 4B). To assess whether this developmental delay is nutrient-dependent, we assessed developmental timing of control and *ceng1A* mutants on different food sources: Musselmann and Palanker described that feeding larvae a food source containing predominantly sugar (HSD, 70% sucrose) causes adipositas-like phenotypes and results in a strong delay in development [26]. In our experiments, we observed a delay of five days in the control animals. Similarly, *ceng1A* mutants show an increase in developmental timing under HSD conditions: Instead of after five to six days, *ceng1A* mutant larvae pupariate after ten to eleven days (Figure 4E). The developmental delay, however, between the mutant and control animals is comparable to the delay under normal fed conditions.

In contrast to the HSD, feeding wildtype animals with a diet composed of mainly fatty acids (HFD, 65% palm fat) does not result in hyperglycemia or increased TAG. Also, developmental timing is not affected in control or *ceng1A* mutant animals compared to the normal fed condition (Figure 4D).

In summary, *ceng1A* mutants show no difference in their response to high sugar or high fat feeding conditions; the onset of pupariation is delayed by one to two days under all conditions.

To examine the developmental delay in *ceng1A* mutants in more detail, we analyzed the growth rate and duration of growth by measuring weight and length from first instar until pupariation (Figure 4F, S4A, B). Weight and length of *ceng1A* mutants is reduced throughout all larval stages. However, with a delay of 8 hours they reach wildtypic weight and length before pupariation (Figure 4F, S4A, B) indicating a reduced growth rate and a longer growth period in the mutants. A closer look at the growth rate graph points towards a mayor growth delay in second instar between 45 and 80 hours after egg deposition. After 80 hours, growth rate of the mutants proceeds in parallel to the controls (Figure 4F, S4A, B). This is consistent with a prolonged second instar stage. In contrast, the duration of L3 stage seems not affected (Figure 4F, S4A, B).

During the *Drosophila* life cycle, embryonic development, larval molts, pupariation and metamorphosis delineate transitions from one developmental stage to the next. These developmental transitions are tightly regulated by pulses of the steroid hormone ecdysone which activates signaling cascades triggering maturation or extending development, depending on nutrient levels and growth status [27], [28].

Ecdysone production in the prothoracic gland is regulated by several factors and pathways including the Prothoracicotropic hormone (PTTH), the insulin and TOR pathway as well as Ras signaling [29]–[32]. Ecdysone activates the ecdysone receptor (EcR), a member of the nuclear receptor family, and its receptor binding partner Ultraspiricle (USP) which form heterodimers to act as transcription factors for target genes like the transcription factors *E74*, *E75* and the *Broad-Complex*. The target genes control the late genes in order to prompt biological changes associated with each ecdysone pulse [33], [34].

To assess whether ecdysone signaling is affected in *ceng1A* mutants, we analysed expression of the ecdysone inducible genes *E75B* and *E74A*, as well as *PTTH* expression from the second instar stage to pupariation. Whereas we could not detect significant changes in *PTTH* expression between *ceng1A* mutants and controls (Figure 3C; S4D, G), we found, however, a reduced induction of the ecdysone targets *E75B* and *E74A* (Figure 4G, S4C, E, F). Induction of *E75B* and *E74A* seem to occur at the correct time points (e.g. 72 or 96 hours after egg deposition), however, the induction is lower than in the control aninmals. At 72 hours after egg lay *E75B* induction is only 0.5 fold compared to the controls (Figure S4E). One peak is even missing (88 hours after egg deposition, Figure 4G). In 76% of the time points, expression of *E75B* is reduced in *ceng1A* mutants compared to the controls (Figure S4E; 53% for *E74A*, Figure S4F). Comparison of growth rate and larval stage with ecdysone peaks (see yellow bars Figure 4F, G) shows a clear correlation of ecdysone peaks and plateau phases in growth of the control animals. At 72 hours and 88 hours after egg lay those coincide with L2/L3 or L3/pupae transition respectively. In the mutants plateau phases in growth e.g. at 72 hours does not coincide with the ecdysone peaks and stage transition. Reduced growth rate as well as transition of larval stages is delayed. In contrast, the duration of the third instar stage is not altered, indicating that the main defect is in L2 larval stage. Of note, similar developmental delay phenotypes are found when ecdysone signaling is decreased [32], [35], [36]. Mutants of the ecdysone target E75A e.g. are stuck in 2nd instar larval stage and pupariate without molting into third instar larvae [37]. Layalle et al. showed previously that via the nutrient sensor AMPK, TOR signaling couples nutrition to developmental timing by modifying the timing of ecdysone peaks in the prothoracic gland in a nutrition-dependent manner [29]. However, we didn't find an effect of Ceng1A on AMPK phosphorylation. Moreover, even though delayed in timing, *ceng1A* mutants still respond normally to different nutrient conditions and the timing of ecdysone peaks themselves is not affected. This argues that coupling of nutritional status to timing is still intact, indicating that TOR signaling is not affected in the mutants. Nor did we find in *ceng1A* mutants significant alterations of the activities of IlS components such as Akt or a change of FOXO target genes or changes in organ size. Therefore, our data point towards a very specific role of CenG1A in ecdysone signaling independent of IlS and TOR/AMPK signaling at the transition from L2 to L3 stage which has to be explored further in the future.

## Conclusions

In summary, in contrast to the *PIKE* -/- knockout mice we did not find a major impact of *ceng1A* on insulin signaling, sensitivity towards starvation or mobilization of lipids under high fed conditions. However, homozygous *ceng1A* mutants show a delay in development specifically in the second larval stage, leading to late onset of pupariation. We found that this delay is nutrition-independent, suggesting a metabolism-independent but ecdysone-dependent mechanism. Together, we propose a novel role of *Drosophila* Ceng1A in regulating developmental timing.

## Supporting Information

Figure S1 Ceng1A is predominantly expressed in the nervous system. Whole-mount in situ hybridizations of wildtype embryos (A-F) and larvae (G,G',H). *ceng1A* expression pattern is visualized by digoxigenin-labelled *ceng1A* antisense RNA probe. Overexpression of *ceng1A* via *pairedGal4* served as a positive (I), *ceng1A* mutants as a negative control (G', J).
(TIF)

Figure S2 Validation of successful homologous recombination by PCR. Utilized primer pairs are indicated in (A). Actin was amplified as a control (A').
(TIF)

Figure S3 AMPK phosphorylation is not affected in *ceng1A* mutants. Quantification (A') of western blots of control and *ceng1A* mutant larvae stained for pAMPK (A). Quantification relative to loading control. n = 3; error bars indicate SEM.
(TIF)

Figure S4 Ceng1A affects growth is reduced in second instar larval stages. From egg deposition to pupariation length (A) and weight (B) of control and *ceng1A* mutant animals was determined every 5 hours. Growth was assessed as an increase of length or weight over time. (A) Throughout larval development, *ceng1A* mutants are smaller than their wildtypic counterparts. (B) Plotting larval length versus larval weight reveals no difference in growth rate between control and *ceng1A* mutant animals. n = 3 for all experiments; error bars indicate SEM. Ceng1A affects expression of ecdysone target genes, but not *PTTH*. From 48 hours after egg deposition to pupariation, expression of the ecdysone target genes *E74A* (C) and *PTTH* (D) was analyzed in control and *ceng1A* mutant larvae via *real-time* RT-PCR. (E – G) Expression of *E75B* (E), *E74A* (F) and *PTTH* (G) in *ceng1A* mutants relative to control indicates that *E75B* and *E74A* are downregulated in most of the time points, whereas *PTTH* expression is not affected. n = 3 for all experiments; error bars indicate SEM.
(TIF)
