# Supplementary figures and images for: The PIKE Homolog Centaurin gamma Regulates Developmental Timing in Drosophila
Source: PLoS One. 2014 May 20;9(5):e97332. doi: 10.1371/journal.pone.0097332 (PMC4028201; doi:10.1371/journal.pone.0097332)

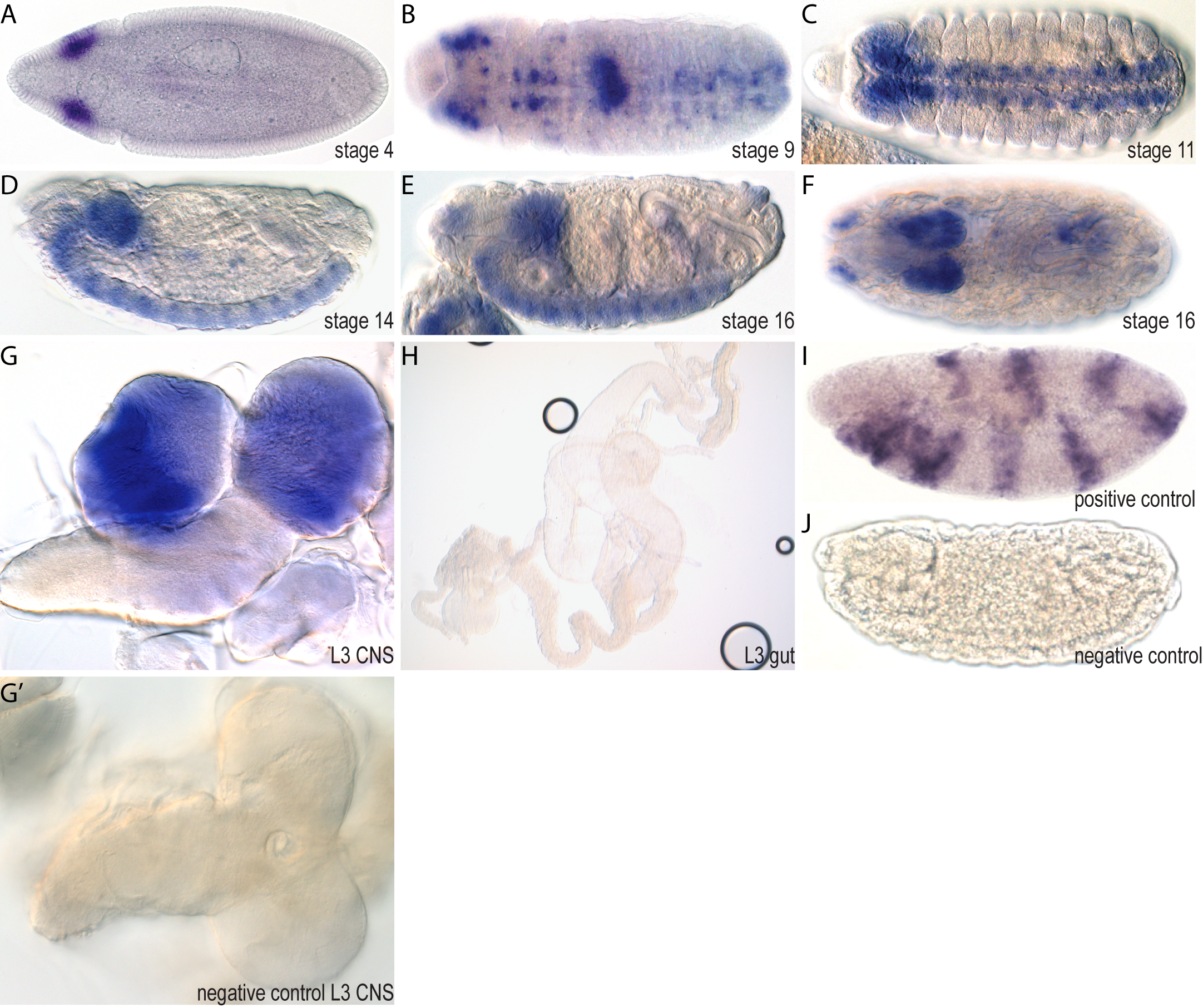

Supplement: Figure S1 — Ceng1A is predominantly expressed in the nervous system. Whole-mount in situ hybridizations of wildtype embryos (A-F) and larvae (G,G',H). ceng1A expression pattern is visualized by digoxigenin-labelled ceng1A antisense RNA probe. Overexpression of ceng1A via pairedGal4 served as a positive (I), ceng1A mutants as a negative control (G', J). (TIF) [file pone.0097332.s001.tif]

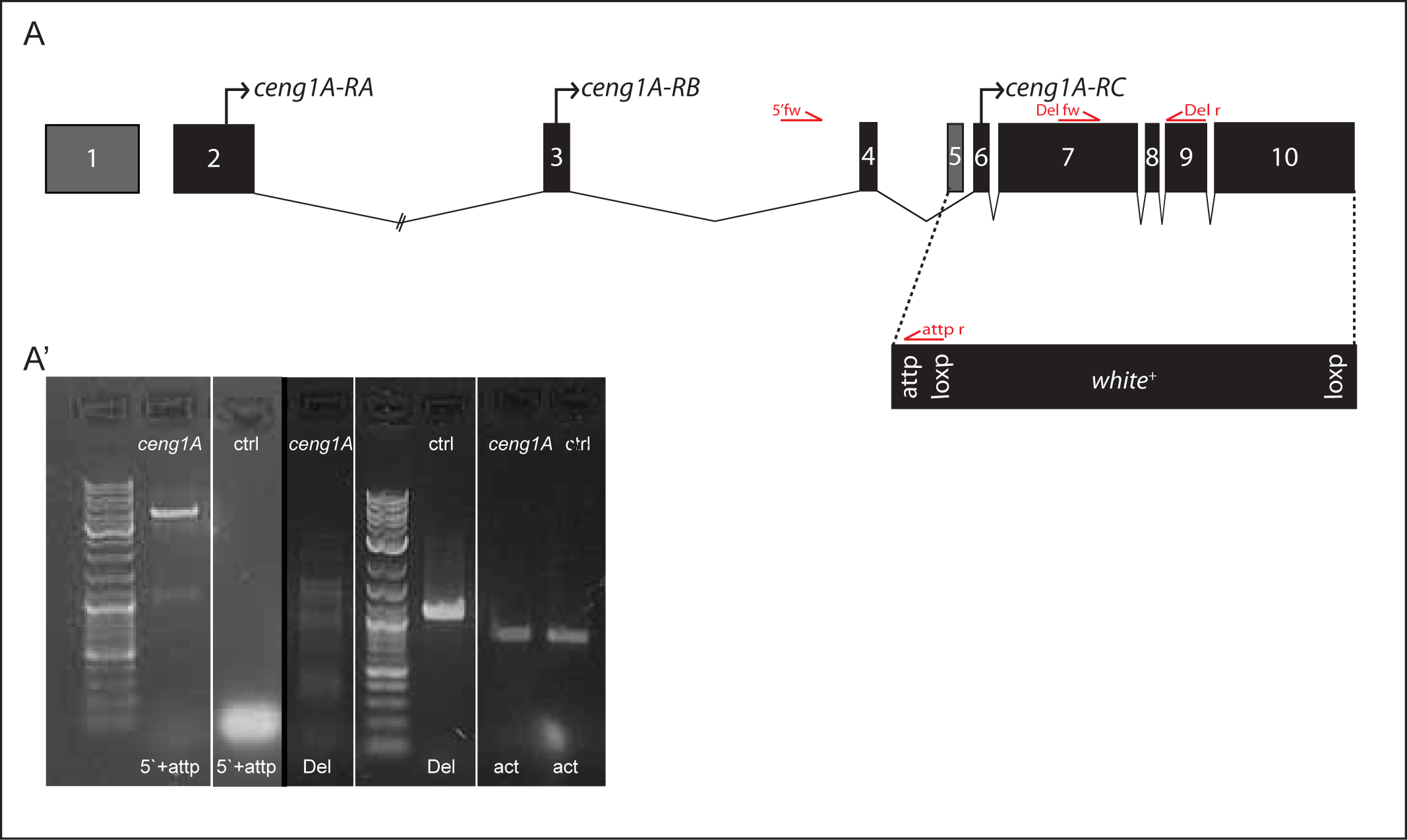

Supplement: Figure S2 — Validation of successful homologous recombination by PCR. Utilized primer pairs are indicated in (A). Actin was amplified as a control (A'). (TIF) [file pone.0097332.s002.tif]

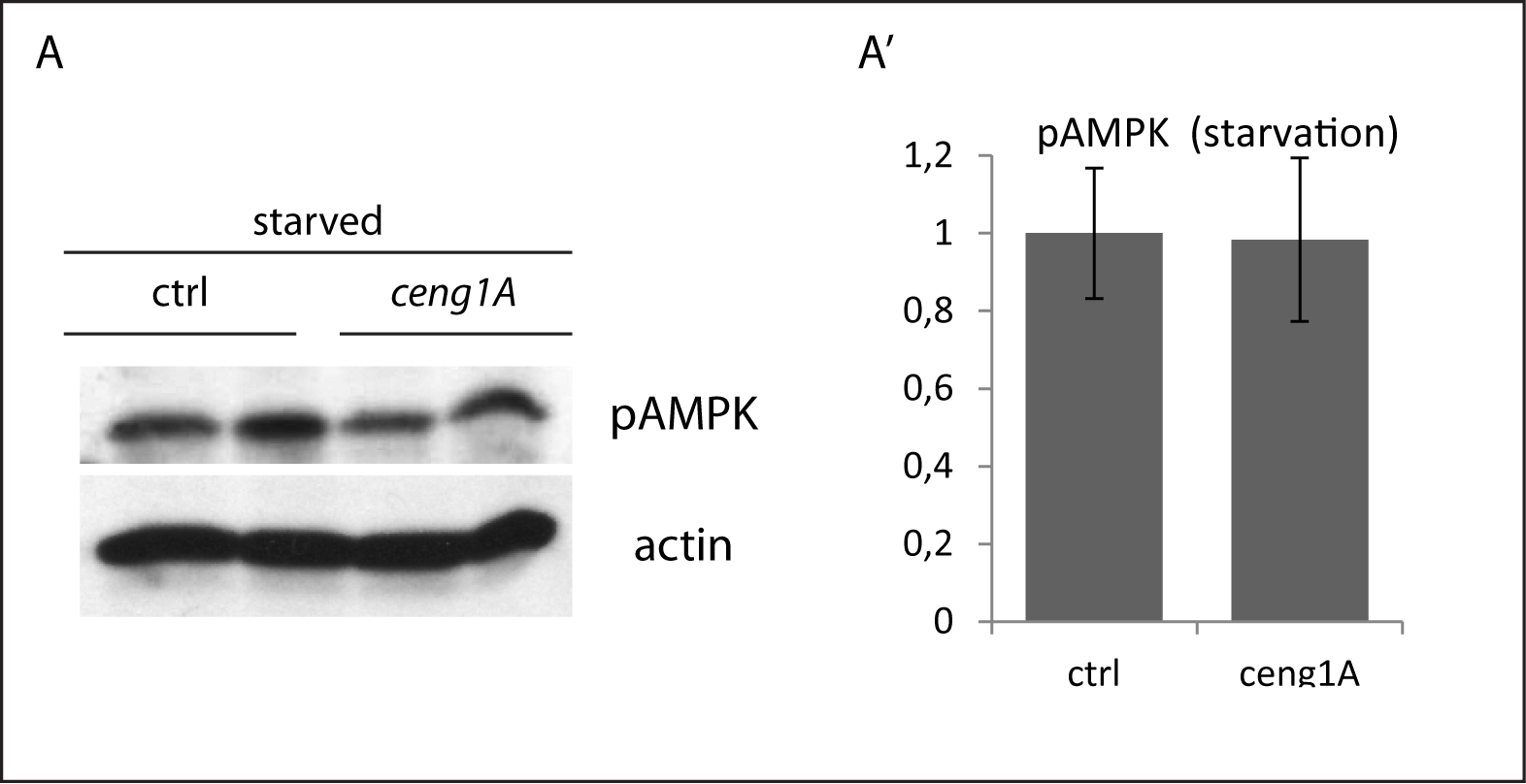

Supplement: Figure S3 — AMPK phosphorylation is not affected in ceng1A mutants. Quantification (A') of western blots of control and ceng1A mutant larvae stained for pAMPK (A). Quantification relative to loading control. n = 3; error bars indicate SEM. (TIF) [file pone.0097332.s003.tif]

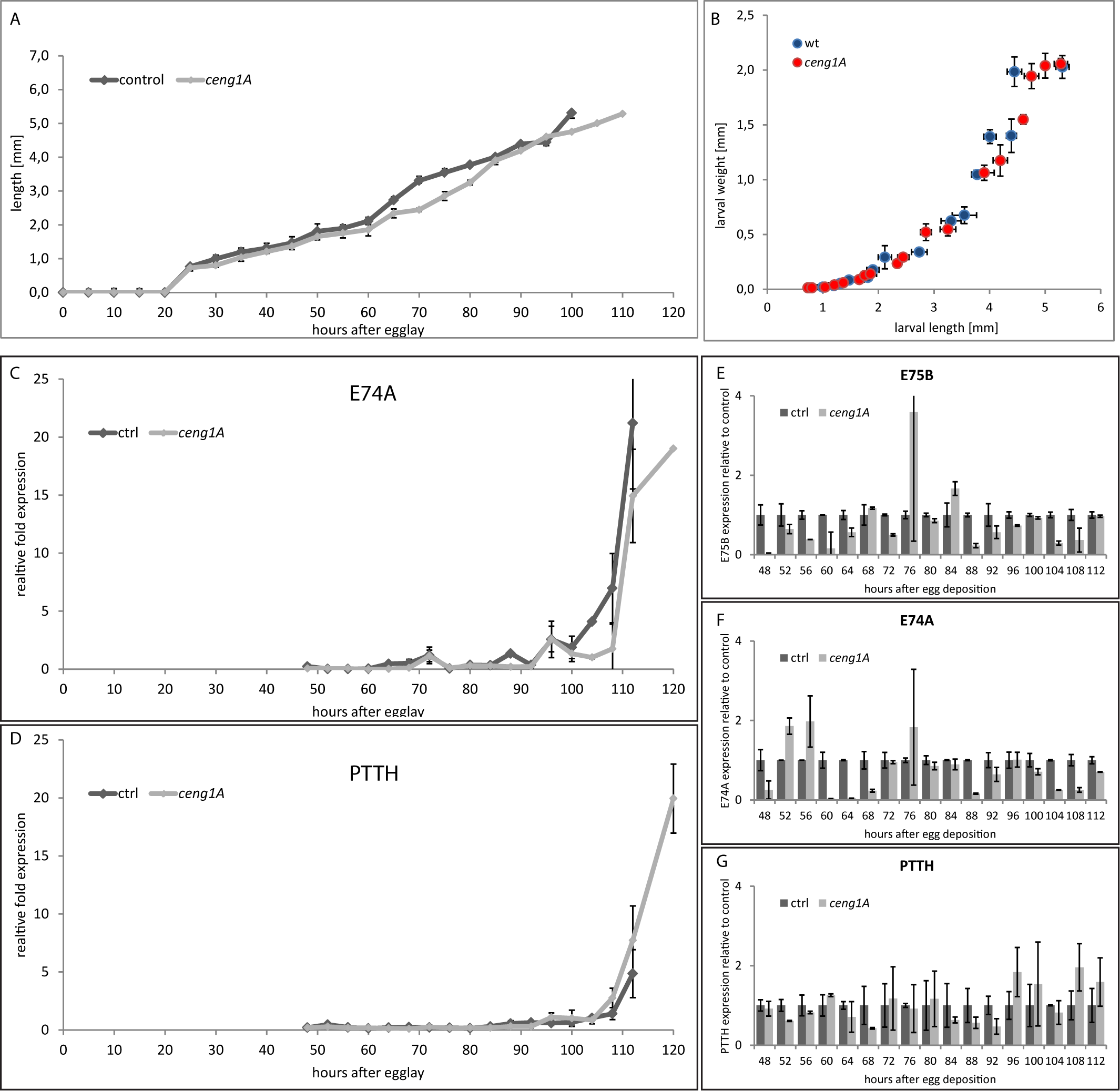

Supplement: Figure S4 — Ceng1A affects growth is reduced in second instar larval stages. From egg deposition to pupariation length (A) and weight (B) of control and ceng1A mutant animals was determined every 5 hours. Growth was assessed as an increase of length or weight over time. (A) Throughout larval development, ceng1A mutants are smaller than their wildtypic counterparts. (B) Plotting larval length versus larval weight reveals no difference in growth rate between control and ceng1A mutant animals. n = 3 for all experiments; error bars indicate SEM. Ceng1A affects expression of ecdysone target genes, but not PTTH. From 48 hours after egg deposition to pupariation, expression of the ecdysone target genes E74A (C) and PTTH (D) was analyzed in control and ceng1A mutant larvae via real-time RT-PCR. (E – G) Expression of E75B (E), E74A (F) and PTTH (G) in ceng1A mutants relative to control indicates that E75B and E74A are downregulated in most of the time points, whereas PTTH expression is not affected. n = 3 for all experiments; error bars indicate SEM. (TIF) [file pone.0097332.s004.tif]
